# Experimental evidence that cuckoos choose host nests following an egg matching strategy

**DOI:** 10.1098/rspb.2022.2094

**Published:** 2023-02-22

**Authors:** Jinggang Zhang, Peter Santema, Zixuan Lin, Lixing Yang, Meijun Liu, Jianqiang Li, Wenhong Deng, Bart Kempenaers

**Affiliations:** ^1^ Ministry of Education Key Laboratory for Biodiversity Sciences and Ecological Engineering, College of Life Sciences, Beijing Normal University, Beijing 100875, People's Republic of China; ^2^ Department of Behavioural Ecology and Evolutionary Genetics, Max Planck Institute for Ornithology, Seewiesen 82319, Germany; ^3^ Edward Grey Institute, Department of Zoology, University of Oxford, Oxford OX1 3PS, UK; ^4^ Academy of Forestry Inventory and Planning, National Forestry and Grassland Administration, Beijing 100714, People's Republic of China; ^5^ School of Ecology and Nature Conservation, Beijing Forestry University, Beijing 100083, People's Republic of China

**Keywords:** brood parasitism, nest selection, egg matching, Daurian redstart, common cuckoo

## Abstract

The arms race between brood parasites and their hosts provides a classic model to study coevolution. Hosts often reject the parasitic egg, and brood parasites should therefore select host nests in which the colour of the eggs best matches that of their own. Although this hypothesis has received some support, direct experimental evidence is still lacking. Here, we report on a study of Daurian redstarts, which show a distinct egg-colour dimorphism, with females laying either blue or pink eggs. Redstarts are often parasitized by common cuckoos, which lay light blue eggs. First, we showed that cuckoo eggs were more similar in spectral reflectance to the blue than to the pink redstart egg morph. Second, we report that the natural parasitism rate was higher in blue than in pink host clutches. Third, we performed a field experiment in which we presented a dummy clutch of each colour morph adjacent to active redstart nests. In this set-up, cuckoos almost always chose to parasitize a blue clutch. Our results demonstrate that cuckoos actively choose redstart nests in which the egg colour matches the colour of their own eggs. Our study thus provides direct experimental evidence in support of the egg matching hypothesis.

## 1. Introduction

Avian brood parasites impose a heavy cost on their hosts in terms of lost reproductive opportunity and misdirected parental care. This selects for defensive host adaptations which, in turn, select for counteradaptations in brood parasites [1]. These reciprocal interactions between brood parasites and their hosts form a classic example of coevolution [2], and have been well studied for more than 30 years [1,3]. However, the question of whether brood parasites parasitize host nests randomly or whether they select nests within a given host population based on characteristics that may influence their reproductive success remains unsolved.

For hosts of parasitic cuckoos, a widespread and effective anti-parasite defence is removing the parasitic egg from the nest [2]. In response to the hosts' egg-rejection behaviour, selection has generally favoured parasitic cuckoos laying eggs that mimic the appearance of host eggs [4–7]. The mimicry, in turn, selects for increased variation in egg appearance among hosts within a population (e.g. colour polymorphism) [5,8,9]. In this way, cuckoo eggs will be more similar to some hosts’ clutches than to others, whereby the less mimetic cuckoo eggs are more likely to be rejected by the host [10]. Following optimality theory [11], cuckoos are then expected to preferentially select host nests in which the colour of the eggs matches their own eggs to maximize their own reproductive success (‘egg matching’ hypothesis) [1,12–14]. Some studies have tested the hypothesis, but they provide mixed evidence.

One study on reed warblers (*Acrocephalus scirpaceus*) [12] and two studies on great reed warblers (*Acrocephalus arundinaceus*) [13,14] compared the match between the egg of the common cuckoo (*Cuculus canorus*) and the host eggs in parasitized and non-parasitized nests. In support of the egg matching hypothesis, all three studies found that cuckoo eggs were more similar to host eggs in naturally parasitized nests than in non-parasitized nests. However, two studies using a similar approach, one on marsh warblers (*Acrocephalus palustris*) [15] and one on oriental reed warblers (*Acrocephalus orientalis*) [16], found no evidence that cuckoo host egg matching was better in parasitized nests than in unparasitized nests.

One problem with such studies is that other explanations for the difference in host–parasite egg similarity between parasitized and non-parasitized eggs cannot be excluded. For instance, hosts are typically more likely to reject a parasitic egg and reject it quicker when it is more dissimilar to their own eggs [17]. Thus, cases of parasitism may more likely go undetected when the match with the host eggs is poor. Moreover, these studies assume that non-parasitized nests had been found by a cuckoo, but that the cuckoo rejected them, i.e. chose not to lay its egg in those nests [12–14].

More recently, Yang *et al*. [18,19] developed a novel experimental method to directly test whether cuckoos select host nests based on egg colour. Yang *et al*. placed clutches of different colours near active nests of several cuckoo host species and found that cuckoos lay eggs randomly with respect to the colour of the host's eggs, thus providing no support for the egg matching hypothesis. Instead, the studies concluded that host activity and nest type are important cues for cuckoo parasitism [18,19]. In sum, there is no direct experimental evidence supporting the egg matching hypothesis, and the correlational support is inconclusive.

Here, we report on an experimental test of the egg matching hypothesis in a population of Daurian redstarts (*Phoenicurus auroreus*), a common host of common cuckoos (hereafter ‘cuckoo’) in northeastern China [20]. Daurian redstarts show a distinct egg-colour dimorphism, with some females laying pink eggs (with all eggs having rusty spots) and others laying blue eggs (with most eggs having rusty spots), whereby the latter appear more similar to the pale blue cuckoo eggs, at least to human eyes (most cuckoo eggs also have rusty spots and lines) [20]. Correspondingly, redstarts laying pink clutches show much higher rejection rates towards both real and model cuckoo eggs than individuals laying blue clutches [20,21]. According to the egg matching hypothesis, cuckoos are thus expected to preferentially select redstart nests with blue eggs to maximize their reproductive success.

In a previous study [20], we found that redstart nests with blue eggs were indeed more often parasitized by cuckoos than nests with pink eggs, but this was based on naturally observed parasitism frequency only. Moreover, we assumed that cuckoo eggs are more similar to redstarts' blue eggs than to pink eggs, but this was based on our subjective assessment [20], which may be unreliable given that avian and human vision systems differ fundamentally, and birds can perceive ultraviolet light [22]. In this study, therefore, we first investigated whether blue redstart eggs are more similar to cuckoo eggs than pink host eggs in avian colour vision terms. Second, based on field observations, we compared natural rates of brood parasitism between blue and pink redstart clutches. Finally, we performed a field experiment in which we presented a dummy clutch of each colour morph close to active redstart nests with blue or pink eggs to test whether cuckoos preferentially select redstart nests with blue eggs.

## 2. Methods

### (a) Study site and species

The study was conducted in ShuangYu, a village in northeastern China (43°37′19″ N, 126°09′54″ E) from 2018 to 2022. Daurian redstarts are common summer residents at the study site, and mainly breed in the vicinity of human habitation. Redstarts generally build nests in enclosed sites and readily use artificial nest-boxes. From 2016 to 2022, 240 nest-boxes were placed in the study site.

Daurian redstarts in our population typically produce two clutches within a single-breeding season. However, owing to the late arrival of the cuckoos, nests are only parasitized during the second egg-laying period (for detailed information, see [20]). The natural parasitism rate therefore strongly varies within a season, with no nests being parasitized in the first egg-laying period (*n* = 207), and 15.6% of nests parasitized in the second laying period (*n* = 358) [20].

### (b) Egg-colour quantification

**Figure 1.**
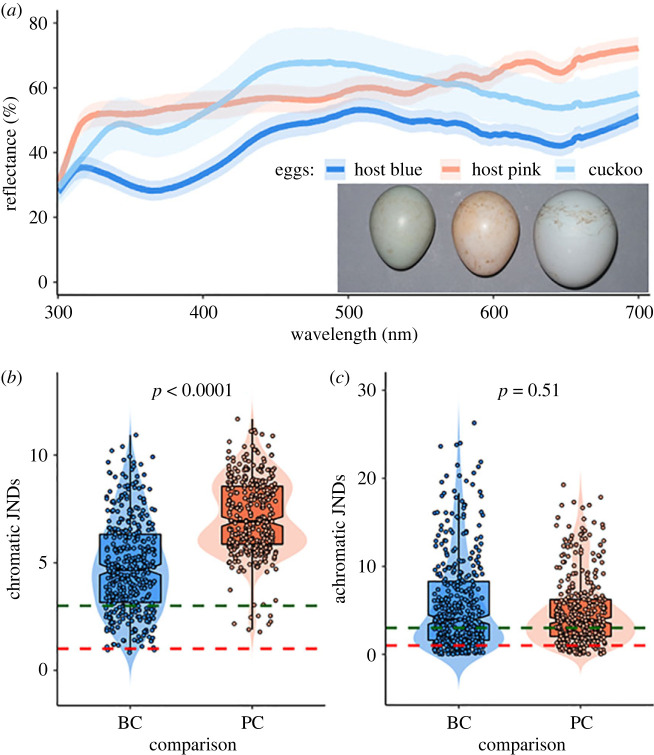
Evidence showing that the colour of common cuckoo eggs is more similar to that of blue Daurian redstart eggs than to that of pink host eggs. (*a*) Reflectance spectra of common cuckoo eggs and blue and pink redstart eggs. Lines and shading indicate means and 95% confidence intervals of reflectance values of 20 cuckoo eggs, 20 blue host clutches and 18 pink host clutches, respectively. (*b*) Chromatic and (*c*) achromatic contrasts (just noticeable differences, JNDs) between cuckoo eggs and redstart eggs of different colour morphs. Shown are the differences between cuckoo eggs and blue redstart eggs (BC) and the differences between cuckoo eggs and pink redstart eggs (PC). Box plots show the median, first and third quartile and 1.5 × interquartile range. Dots show raw data generated from receptor noise limited (RNL) models (see Methods for details). Each data point shows the contrast between one redstart clutch (average values) and one cuckoo egg. Smaller JND values indicate a closer colour match between cuckoo and redstart eggs. The dashed lines denote two JND thresholds: data points below the green line (JND = 3) indicate that birds may be able to distinguish the difference with difficulty, and data points below the red line (JND = 1) indicate that the difference cannot be perceived by birds. *p*-values are based on Mann–Whitney *U* tests, comparing all possible pairs of cuckoo eggs and blue, respectively pink, redstart eggs. Only results from the ultraviolet-sensitive bird vision system are presented.

In 2021, we measured both cuckoo eggs (*n* = 20) and redstart eggs using a spectrometer (AVANTES 2048, Avantes, Apeldoorn, The Netherlands) to obtain reflectance spectra in the 300–700 nm range (figure 1*a*). In each of 38 redstart clutches (20 clutches of blue eggs and 18 clutches of pink eggs), we randomly selected and measured three eggs (clutch size in the second egg-laying period: mean ± s.d. = 5.6 ± 0.8, *n* = 534). For each egg, we divided its surface into three regions across the longitudinal axis (blunt end, middle, and sharp end) and took three measurements (each covering *ca* 1 mm^2^) from each region, avoiding small spots of a different colour. We did not consider egg-spot patterns, because the three types of eggs (cuckoo egg, blue host egg and pink host egg) can be easily distinguished based on the background colour (figure 1*a*).

Before analysis, we averaged all nine measurements (3 measurements × 3 regions) from an egg, and then averaged data of the three eggs within a clutch. Following [14], we calculated the chromatic and achromatic contrasts between cuckoo eggs and the two types of Daurian redstart eggs using Vorobyev & Osorio's receptor noise limited (RNL) models implemented in AVICOL v.6 [22–24]. These models integrated the reflectance spectra of cuckoo eggs and two types of Daurian redstart eggs, ambient light conditions (irradiance spectra inside a cavity for a hole-nesting species such as the Daurian redstart were taken from [25]), photoreceptor noise and single- and double-cone photoreceptor spectral sensitivities [26]. Although spectral sensitivity has never been measured in the cuckoo, most birds have one of the two types of vision systems: ultraviolet-sensitive (UVS) or violet-sensitive (VS) [27]. Therefore, we ran two models, one using data from a representative of the UVS type of vision (the blue tit, *Cyanistes caeruleus*; proportions of single cones: UVS single cones = 1, short-wavelength-sensitive (SWS) single cones = 1.92, medium-wavelength-sensitive (MWS) single cones = 2.68 and long-wavelength-sensitive (LWS) single cones = 2.70, derived from [28]) and one with data from a representative of the VS vision system (the Indian peafowl, *Pavo cristatus*; proportions of single cones: UVS = 1, SWS = 1.9, MWS = 2.2 and LWS = 2.1, derived from [29]), and conducted statistical analyses for both vision types separately. This procedure generated estimates of both chromatic (difference in hue) and achromatic (difference in brightness) contrasts between cuckoo eggs and two types of redstart eggs in just-noticeable-difference (JND) units. Colour discrimination thresholds might vary throughout colour space (as shown in fish [30]), but behavioural experiments would be needed to determine the discrimination thresholds of cuckoos. Because such experiments are beyond the scope of this study, we interpreted the results using a more conservative approach compared with other studies [31,32], i.e. JND values ≤ 1 imply that birds cannot perceive the difference, JND values ≤ 3 imply it is difficult for birds to distinguish the difference, and larger JND values correspond to larger differences between the two egg spectra as perceived by birds [15,22,33].

### (c) Field observations

During each breeding season, we searched for nests in natural or semi-natural cavities (e.g. in buildings) every day and checked empty nest-boxes 1–2 times per week. When a nest (either natural or in a nest-box) was found during the nest-building stage, we checked it every 1–2 days to determine the laying date and we recorded clutch colour. After cuckoos arrived at the study site, we checked each nest in the egg-laying or early incubation stage every afternoon to assess the presence of a parasitic egg. Cuckoo eggs are easily distinguished from redstart eggs by both size and colour (figure 1, and Fig. 1 in [20]). During previous artificial parasitism experiments, we had found some cuckoo egg models that were rejected by the host on the ground near the nest. We therefore also checked the ground surrounding the nest to investigate the possibility that a cuckoo egg was rejected by the host before our nest visit. However, we never found a cuckoo egg on the ground during the study. During the second egg-laying period from 2018 to 2020, we monitored 561 nests, but 83 of these nests were found during the nestling stage such that we did not have information on clutch colour. The number of nests included in the analyses was therefore 478.

### (d) Cuckoo choice experiment

**Figure 2.**
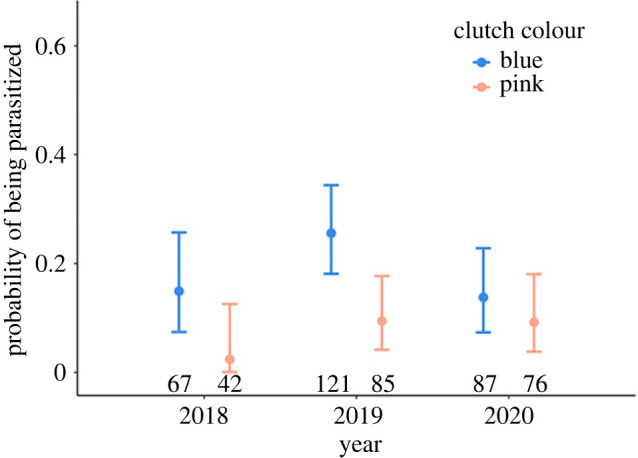
Cuckoo parasitism probabilities for Daurian redstart nests with blue and pink clutches in three years without experimental manipulations. Points and error bars indicate means and 95% confidence intervals generated with the R function *binom.test* based on the raw data. Numbers above the *x*-axis indicate sample sizes.

During the second egg-laying period in 2021 and 2022, we performed an experiment to test whether cuckoos preferentially parasitize a nest based on host clutch colour. Following the experimental paradigm described in [18], we placed two artificial redstart nests, one with blue eggs and one with pink eggs, near each of 134 active redstart nests (2021: *n* = 68, 2022: *n* = 66), of which 81 had blue eggs and 53 had pink eggs. We only used nests in nest-boxes for this experiment. On the day we found the first egg in an active nest, we placed two old but complete nests (in nest-boxes, collected during the first egg-laying period) close to the active nest (figure 2). Because nest-boxes were placed either on a power pole or on a building, the three experimental nest-boxes were placed vertically or horizontally, respectively. The relative positions of the three nests were decided randomly, but the distance between any two adjacent nests was less than 50 cm to minimize variation in host activity near the nests, which may influence cuckoo nest selection [19]. Every morning (8.00–9.00) during the egg-laying stage, we placed a real redstart egg (collected from previous clutches that had failed) with the same or a different colour morph in the two dummy nests, such that the clutch size matched that of the focal active nest. We also checked all active and dummy nest-boxes (and the surrounding ground) for the presence of cuckoo eggs, every morning and every afternoon (after 17.00), to maximize the chances of detecting cuckoo parasitism. All nest-boxes involved in the experiment were checked until the fourth day after incubation in the active nest.

### (e) Statistical analyses

We compared the chromatic and achromatic JNDs between cuckoo eggs and blue host eggs and between cuckoo eggs and pink host eggs using Mann–Whitney *U* tests, because of the non-normal distribution of the data. The comparisons were based on all possible pairs between cuckoo eggs and blue or pink redstart eggs.

We used a chi-squared test to compare the naturally observed cuckoo parasitism frequency between nests with a blue clutch and nests with a pink clutch (data from 2018 to 2020, only the second egg-laying period considered).

We investigated whether cuckoos were more likely to parasitize a nest of an experimental triplet based on (i) nest type (active or dummy) and (ii) egg-colour morph (blue or pink). Owing to the small sample sizes, we used Fisher's exact tests to compare the frequency of brood parasitism between groups for triplets with a blue or pink active clutch, respectively.

All statistical analyses were conducted in R v.3.6.3 [34]. Values are shown as mean ± s.d. The alpha level was set at 0.05.

## 3. Results

### (a) Egg-colour comparisons

The chromatic contrast (JND) between cuckoo eggs and blue redstart eggs was lower than that between cuckoo eggs and pink redstart eggs (table 1; figure 1*b*), but there was no significant difference in achromatic contrast (table 1; figure 1*c*). The colour (i.e. hue, but not brightness) of cuckoo eggs is thus more similar to that of blue redstart eggs than to the colour of pink redstart eggs. However, there is still an easily noticeable difference between cuckoo eggs and host blue eggs, as cuckoo eggs are lighter (figure 1*a*) and both the chromatic and achromatic JNDs are larger than 3 (table 1; figure 1*b,c*).

**Table 1.** Results from Mann–Whitney *U* tests comparing colour differences between common cuckoo eggs and blue or pink Daurian redstart eggs.

vision system	contrast	comparison	JND value	*U*	*p*
UVS	chromatic	cuckoo eggs versus blue host eggs	4.8 ± 2.2	29 848	<0.0001
		cuckoo eggs versus pink host eggs	7.1 ± 1.8		
	achromatic	cuckoo eggs versus blue host eggs	5.7 ± 5.4	74 004	0.51
		cuckoo eggs versus pink host eggs	4.9 ± 3.9		
VS	chromatic	cuckoo eggs versus blue host eggs	3.1 ± 1.3	10 082	<0.0001
		cuckoo eggs versus pink host eggs	6.2 ± 1.8		
	achromatic	cuckoo eggs versus blue host eggs	5.6 ± 5.3	73 155	0.7
		cuckoo eggs versus pink host eggs	4.9 ± 3.9		

### (b) Observed cuckoo parasitism and clutch colour

Of 561 active redstart nests during the second egg-laying period, 80 (14.3%) were parasitized by cuckoos. Blue-egg nests showed a significantly higher probability of cuckoo parasitism (19.3%, *n* = 275) than pink-egg nests (7.9%, *n* = 203; *χ*^2^ = 9.34, d.f. = 1, *p* = 0.002; figure 2).

### (c) Cuckoo choice experiment

**Figure 3.**
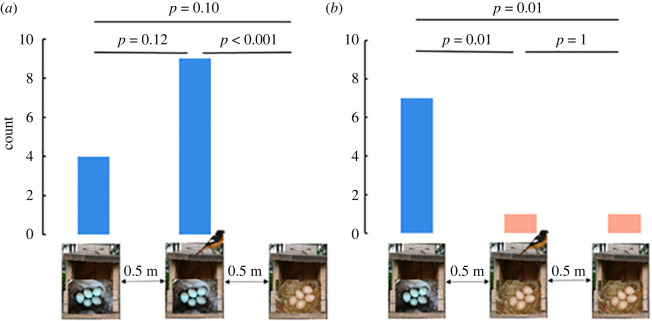
Illustration of the experimental design (the experimental triplet with a blue (*a*) or a pink active Daurian redstart clutch (*b*)) and the results of the cuckoo choice experiment showing that blue host clutches are more likely to be parasitized by cuckoos than pink host clutches. Each experimental triplet consisted of one active redstart nest (middle, with bird) and two dummy nests, one with blue (left) and one with pink eggs (right). Note that in the field, the three nest-boxes of a triplet were randomly ordered, i.e. the active nest was not always in the middle. Bars indicate the number of nests parasitized by a cuckoo. In each triplet, only one nest was parasitized. *p*-values above bars are based on Fisher's exact tests.

Of 134 trials (with one active and two dummy nest-boxes each), 22 triplets (16.4%) were parasitized by a cuckoo. Of the 13 parasitized triplets with a blue active redstart clutch, all cuckoo eggs were found in nests with blue eggs (9 and 4 in active and dummy nests, respectively). Of the nine parasitized triplets with a pink active clutch, 7 cuckoo eggs were found in blue-egg dummy nests, 1 in a pink-egg active nest and 1 in a pink-egg dummy nest (figure 3). The probability that a nest was parasitized did not depend on nest type (significance level between blue-egg active and dummy nests and between pink-egg active and dummy nests is 0.12 and 1, respectively; figure 3), but depended on clutch colour, with blue clutches more likely containing a cuckoo egg than pink clutches (significance level between blue-egg active nests and pink-egg dummy nests *p* < 0.01, and between pink-egg active nests and blue-egg dummy nests *p* = 0.01; figure 3), and the pattern was consistent across years (electronic supplementary material, table S1).

## 4. Discussion

Using spectrometry data, results from avian visual models show that the blue eggs of Daurian redstarts are more similar to cuckoo eggs than redstarts' pink eggs. However, cuckoo eggs are lighter and still noticeably different from Daurian redstart blue eggs. Nevertheless, redstart females laying blue eggs often accept the cuckoo egg [20], which might be because of the dim light in the nest cavity [35]. The hosts’ low egg-rejection frequency may impose little selection on cuckoos to improve egg mimicry. This is, however, apparently not the case in common redstarts (*Phoenicurus phoenicurus*), another known cavity-nesting cuckoo host, because the cuckoo eggs mimic common redstart eggs perfectly in colour [36].

We found that cuckoos were more likely to parasitize redstart nests with blue eggs than those with pink eggs, both when considering the proportion of naturally parasitized nests, and in the cuckoo choice experiment. This suggests that female common cuckoos select host nests based on egg phenotype. Specifically, cuckoos preferentially parasitized redstart nests with blue eggs, which match the colour of their own eggs more closely. As redstarts laying pink eggs are more likely to reject both real and model cuckoo eggs than individuals laying blue eggs [20,21], cuckoos thus effectively reduce the probability that the host will reject their egg. In triplets with a blue active redstart clutch, the active nest was more often parasitized than the blue dummy nest (figure 3*a*). Although this effect is not statistically significant, which may be because of the small sample size, it suggests that activity at the nest also influences cuckoo nest selection. However, in triplets with a pink active clutch, almost all cuckoo eggs (7/9) were found in blue dummy nests (figure 3*b*), suggesting that clutch colour plays a more important role in cuckoo nest selection than activity at the nest.

Previous studies testing the egg matching hypothesis yielded mixed results, with some showing support [12–14,37], but others not [15,16,18,19,38]. Importantly, the two studies that tested the hypothesis experimentally found no supportive evidence [18,19]. For example, the parasitic plaintive cuckoo (*Cacomantis merulinus*) and its host the common tailorbird (*Orthotomus sutorius*) both lay dimorphic eggs, and tailorbirds reject almost 100% non-matching foreign eggs, while accepting all matching eggs [18]. Yet, both naturally observed brood parasitism and a cuckoo nest choice experiment suggest that plaintive cuckoos do not preferentially parasitize host nests that match the colour of their own eggs [18]. One potential explanation for the apparent discrepancy in cuckoo choosiness between studies is the difference in host nest availability [14,37,39]. Indeed, Antonov *et al*. [15] suggested that cuckoos may not select on the appearance of marsh warbler clutches because the hosts breed in low density, such that an increase in search time may offset any benefit of being selective. By contrast, in the studies that suggested cuckoo choosiness in great reed warblers, breeding density was high during the peak breeding period, which may allow cuckoos to be more selective [14,40]. This interpretation is also consistent with our study. During the second egg-laying period, we found about 250 host nests, and based on field observations of cuckoos and the phenotype of their eggs, we estimated that there were about 10 to 15 individual cuckoos present. If this estimate is correct, it indicates that cuckoos have ample choice.

One can argue that the cuckoo choice experiment itself would attract cuckoos and offer them more opportunities to select than under natural circumstances. Indeed, our experimental set-up of triplets of nests does not represent a scenario that cuckoos would naturally encounter. However, Daurian redstarts breed at high density in the study area, and both nest-boxes and natural nests are conspicuous and easy to find, suggesting that cuckoos also have ample choice in nature (as mentioned above). Also, the fact that cuckoos appear to preferentially parasitize blue clutches over pink clutches in a natural setting suggests that they have the ability to choose between clutches (figure 2). Moreover, the experimental set-up itself does not seem to have attracted cuckoos, since the parasitism rate of nests in the cuckoo choice experiment (16.4%) was similar to that of natural nests (14.3%).

To choose a nest with eggs matching in colour, cuckoos are expected to know the appearance of their own eggs. Some studies have suggested that egg recognition in birds is based on an internal template of their own eggs [41–43] and cuckoos may also possess such an ability, although direct evidence is lacking. Alternatively, cuckoo females may learn the colour of their eggs after they lay their first egg, as has been suggested for some host species [44–46]. In this scenario, parasitism in non-matching host nests should be more common in the early breeding season, when inexperienced cuckoos have not yet learned the appearance of their own eggs. Our observations contradict this idea, because nests with pink clutches are parasitized also later in the breeding season. Moreover, the colour contrast between the eggs and the nest background may also be important for egg recognition by both hosts and brood parasites [47,48]. Thus, the colour contrast between Daurian redstart eggs and the nest background may also affect the cuckoos' choosiness, but unfortunately we cannot test this with our data because we did not measure the colour of the nest cup.

Alternatively, the cuckoos’ preference for blue-egg redstart nests could have evolved even if cuckoos do not know the appearance of their own eggs. Given that redstarts laying pink eggs consistently show a higher rejection rate towards cuckoo eggs than redstarts laying blue eggs [20,21,49], selection could have favoured cuckoos that preferentially parasitize blue clutches. This would only require that cuckoos can differentiate between host egg colour and does not require the knowledge that their own eggs are blueish. Note that this applies generally to hosts with a distinct egg-colour polymorphism, but not to cuckoo host systems in which the host eggs show subtle but continuous variation in phenotype. For example, in marsh warblers, interclutch variation in egg appearance is more continuous, and the egg phenotypes of the cuckoo gentes vary across the host's continuum [50]. In such systems, knowledge of the appearance of their own eggs and a more subtle recognition ability of the host egg phenotype are required for cuckoos to choose host nests based on egg matching (as discussed in [15]). Thus, the difference between subtle, continuous variation in egg colour compared with a distinct colour polymorphism might explain the discrepancy between our and previous studies ([15,16,19], but see [18]).

Cuckoos may also select blue clutches for reasons other than egg matching. For example, it has been suggested that blue (or blue–green) eggs indicate better female body condition, immunocompetence or oxidative status [51–55], or that it predicts a better nutritional environment [56] or higher parental investment [57]. If this would be the case, cuckoos might benefit from selecting host nests with blue eggs (compared with pink eggs) because their chicks would be raised in a better environment and thus have a higher probability of fledging [58]. This is in line with the ‘optimal egg-laying strategy’, which proposes that when there are plenty of nests available in a given host population, female cuckoos should choose the nest of the highest-quality hosts [40,59]. However, redstarts laying blue eggs did not outperform individuals laying pink eggs in terms of egg volume, clutch size and the proportion of nests that were successful (i.e. that produced at least one fledgling; success rate of blue and pink redstart clutches was 0.45 (95% confidence interval: 0.39–0.51; *n* = 275) and 0.46 (95% confidence interval: 0.39–0.53; *n* = 214), respectively; *χ*^2^ = 0.02, d.f. = 1, *p* = 0.89), and—for those that were successful—the number of fledglings (electronic supplementary material, table S2).

The evolution of egg-colour polymorphisms is often interpreted as an anti-parasite adaptation by cuckoo hosts, because it significantly reduces the success of cuckoo parasitism [10,60]. In this context, the egg-colour dimorphism in Daurian redstarts and the selective parasitism of blue clutches by the common cuckoo can be seen as part of an ongoing coevolutionary process in this host–parasite system. The cuckoos' egg matching strategy may now impose stronger selection on redstarts laying blue eggs than on those laying pink eggs, driving them to improve their egg recognition abilities or to change egg background colour and spotting pattern, and ultimately leading to increased between-individual variation [1]. In the field, we indeed observe some evidence for increased variation in spotting patterns: while both blue and pink redstart eggs typically have rusty red spots, only blue clutches sometimes lack spots altogether. Moreover, although blue redstart eggs are more similar to cuckoo eggs than pink host eggs, many blue clutches were as distinct from cuckoo eggs as the pink eggs (figure 1*b*), which suggests that selection might have led to an increase in between-clutch variation in colour in redstarts laying blue eggs.

In conclusion, our data demonstrate that common cuckoos preferentially parasitize Daurian redstart nests with blue eggs that more closely match their own eggs in colour. By doing so, they can effectively reduce the probability of egg rejection by the host and thus enhance their reproductive success. Our study provides the first experimental evidence of the egg matching hypothesis.

## Data Availability

Raw reflectance spectra of eggs are provided in the electronic supplementary material; data for cuckoo choosiness are available from the Dryad Digital Repository: https://doi.org/10.5061/dryad.5dv41ns8v [61]. Supplementary material is available online [62].
